# Identification of Potential Key Genes and Pathways in Early-Onset Colorectal Cancer Through Bioinformatics Analysis

**DOI:** 10.1177/1073274819831260

**Published:** 2019-02-20

**Authors:** Bin Zhao, Zulqarnain Baloch, Yunhan Ma, Zheng Wan, Yani Huo, Fujun Li, Yilin Zhao

**Affiliations:** 1 Medical College of Xiamen University, Xiamen, Fujian, China; 2 College of Veterinary Medicine, South China Agricultural University, Guangzhou, China; 3 The Department of Anesthesiology, the First Affiliated Hospital of Harbin Medical University, Harbin, Heilongjiang, China; 4 Department of Oncology and Vascular Interventional Radiology, Zhongshan Hospital affiliated of Xiamen University, Xiamen, Fujian, China

**Keywords:** colorectal cancer, bioinformatics analysis, differentially expressed genes

## Abstract

This study was designed to identify the potential key protein interaction networks, genes, and correlated pathways in early-onset colorectal cancer (CRC) via bioinformatics methods. We selected microarray data GSE4107 consisting 12 patient’s colonic mucosa and 10 healthy control mucosa; initially, the GSE4107 were downloaded and analyzed using *limma* package to identify differentially expressed genes (DEGs). A total of 131 DEGs consisting of 108 upregulated genes and 23 downregulated genes of patients in early-onset CRC were selected by the criteria of adjusted *P* values <.01 and |log2 fold change (FC)| ≥ 2. The gene ontology functional enrichment analysis and the Kyoto Encyclopedia of Genes and Genomes (KEGG) pathway analysis were accomplished to view the biological process, cellular components, molecular function, and the KEGG pathways of DEGs. Finally, protein–protein interactions (PPIs) were constructed, and the hub protein module was identified. Genes such as *ACTA2*, *ACTG2*, *MYH11*, *CALD1*, *MYL9*, *TPM2,* and *LMOD1* were strongly implicated in CRC. In summary, in this study, we indicated that molecular mechanisms were involved in muscle contraction and vascular smooth muscle contraction signaling pathway, which improve our understanding of CRC and could be used as new therapeutic targets for CRC.

## Introduction

Colorectal cancer (CRC) is one of the most common malignant diseases in the world, and its incidences increased with age. According to estimates, more than 777 000 of new cases with CRC were registered in 2015 in the developed countries,^1,2^ there were about 376 000 of new CRC cases and 191 000 of death were reported in 2015 in China.^3^ Most CRC were related to old age and lifestyle factors, with only a fraction of cases caused by underlying genetic disorders.^4,5^ Although numerous efforts has been taken to understand the genetic mechanism for initiation and progression of CRC, it remains a major challenge for researchers to prevent and treat early-onset CRC. Therefore, it is important and urgent to uncover the mechanisms of early-onset CRC and develop novel therapeutic routes.

Gene chip or expression profile is a gene-level detection technique that has been applied to scientific research during 2000. Using gene chips, integrated bioinformatic knowledge makes it possible to detect the expression of the entire genome within the same sample in a single experiment, which is particularly suitable to screen differentially expressed genes.^6,7^ With the application of the gene chips, a large amount series of correlated CRC slice data have been produced, archived, and deposited in public databases. Reanalyzing and reintegrating those data sets may find some meaningful clues for new research. A series of microarray data sets have been carried out on CRC in recent years,^8,9^ and a large number of differentially expressed genes (DEGs) have been obtained, which are involved in different pathways, biological process, cellular components, or molecular functions.

In this study, we downloaded the original raw data set (GSE4107) from the website of Gene Expression Omnibus (GEO, http://www.ncbi.nlm.nih.gov/geo/), which is a public database for archiving and querying microarray data. Gene expression profiles of patients with CRC were compared to those in normal healthy control to identify the DEGs. Subsequently, the DEGs were screened using Rstudio software installed Limma packages^10,11^; then gene ontology (GO) and pathway enrichment analysis were performed on the online website DAVID (https://david.%20ncifcrf.gov).^12^ Through analyzing their biological functions and pathways, we may sketch out the outline of CRC development at molecular level and identify the potential candidate genes for diagnosis, prognosis, and therapeutic targets.

## Materials and Methods

### Microarray Data

Microarray data GSE4107^13^ were downloaded from the National Center Biotechnology Information Gene Expression Omnibus (https://www.ncbi.nlm.nih.gov/geo/) database,^14,15^ which was executed with help of GPL570 Human Genome U133 Plus 2.0 Array. GSE4107 contains 12 patients and 10 healthy control (average age: 50 or less, ethnicity: Chinese).

### Data Preprocessing

After GSE4107 was downloaded, probe identification numbers were transformed into gene symbols. For multiple probes corresponding to one gene, the significant expression value was taken as the gene expression value. After that, gene expression values were normalized using the Affy package.^16^

### Identification of DEGs

The raw data GSE4107 files used for analysis included the ***.CEL*** format files (Affymetrix). The analysis was processed and executed by Rstudio software and limma package.^17,18^ We applied linear models for the assessment of differential expression and the analysis of designed experiments.^18,19^ Limma package in Rstudio was applied to identify the DEGs between early-onset CRC samples and healthy control samples. Genes with |log2 fold change (FC)| ≥ 2 and adjusted *P* values <.01 as the cutoff criterion were selected for subsequent analysis.

### Gene Ontology and Pathway Enrichment Analysis

The GO analysis is a useful method for annotating gene and gene product^20^ and identifying characteristic biological meaning of genome and transcriptome.^21,22^ The KEGG is a systematic analysis database of gene function, linking genomic information with high-level functional information.^23^ Candidated DEGs functional-level enrichment were analyzed through multiple online tools. DAVID, among them, is an online website with gene annotation, visualization, and providing gene attributes. *P* < .05 as the cutoff criterion was considered statistically significant.^22,24,25^

### Integration of Protein–Protein Interaction Network and Module Analysis

First, Search Tool for the Retrieval of Interacting Genes (STRING)^26^ database was used to demonstrate DEG-encoded proteins and protein–protein interaction (PPI) information. Second, to evaluate the interactive relationships among DEGs, we mapped the DEGs to STRING, and minimum required interaction score >0.400 (medium confidence) was selected as significant threshold. Then, PPI networks were constructed using the Cytoscape software.^27^ The plug-in Molecular Complex Detection (MCODE), a well-known automated method to find highly interconnected subgraphs as molecular complexes or clusters in large PPI networks, was used to screen the modules or clusters of PPI network in Cytoscape. The MCODE parameters criteria were set by default, except K-core = 6. Moreover, the functional enrichment analysis was performed for DEGs in the modules with *P* < .05 as the cutoff criterion.

## Results

### Identification of DEGs

In this study, we included 12 patients with CRC and 10 healthy controls for the analysis. GSE4107 was analyzed using Rstudio software and following identifies the DEG sets. Using adjusted *P* values <.01 and |log2 fold change (FC)| ≥ 2 criteria, a total of 131 genes were identified; among them, 108 were upregulated and the other 23 were downregulated (Table 1).

**Table 1.** 131 Differentially Expressed Genes (DEGs) Were Identified From GSE4107, Including 108 Upregulated Genes and 23 Downregulated Genes in the Patients With Early-Onset Colorectal Cancer, Compared to Healthy Control.^a^

DEGs	Gene Name
Upregulated	*CYR61, FOS, EGR1, DUSP1, ADAMTS1, VIP, CTGF, MGP, SRPX, LINC01279, FOSB, CCDC80, TSC22D3, RSPO3, RGS1, RHOB, DPT, UCHL1, IGFBP5, ADH1B, FBLN1, PMP22, **ACTA2**, NR4A1, SFRP2, OGN, PLN, PTGIS, CHRDL1, C11orf96, GEM, TIMP3, TAGLN, GREM1, C7, SCGN, DCN, TNS1, ANK2, FILIP1L, ASPN, PTRF, GAL, FABP4, AOC3, FAM129A, RTN1, FHL1, SYNPO2, TUBA1A, CRYAB, RERGL, MFAP5, MAP1B, **MYH11**, GUCY1B3, CXCL12, CLU, AKAP12, TGFB1I1, APOLD1, **MYL9**, DDR2, SELM, MLLT11, RGS2, ATF3, CAV1, **LMOD1**, TMEM47, TUBB6, **ACTG2**, PEG3, COL12A1, ZAK, EBF1, **TPM2**, MAMDC2, SYNM, SCG2, C2orf40, FERMT2, SOCS3, SDPR, SIK1, MSRB3, SCN7A, GUCY1A3, SORBS1, PCDH7, ATP1A2, CNN1, CXCR4, MYOCD, **CALD1**, FLNA, GAS1, CDH19, HSPB6, REEP1, GPM6B, NEXN, KCNMB1, FN1, MEIS2, PGM5, PI15, HSPB8*
Downregulated	*NR1H4, SLC51A, SCIN, NETO2, ETNK1, SLC38A4, HOOK1, GBA3, MEP1B, ACOX1, METTL7B, NQO1, VAV3, CWH43, AKR1C3, DSC2, BCL2L15, EHF, LRRC31, LIMA1, MGST1, UGT2A3, ACE2*

^a^The upregulated genes were listed from the largest to the smallest of fold changes, and downregulated genes were listed from the smallest to largest.

### Gene Term Enrichment Analysis

**Figure 1.**
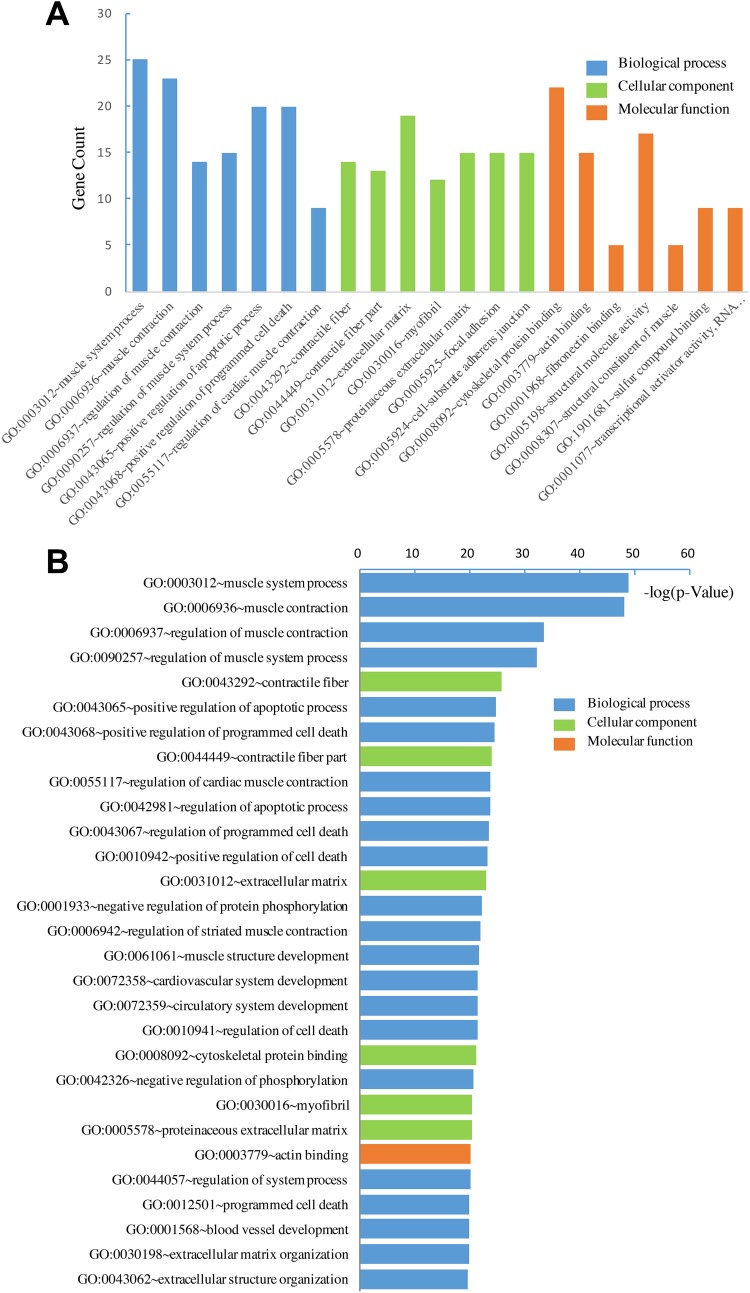
Gene ontology analysis and significant enrichment of differentially expressed genes (DEGs) in early-onset colorectal cancer (CRC). (A) Gene ontology (GO) analysis classified DEGs into BP, CC, and MF group. (B) Ranking significant enriched GO terms of DEGs.

We uploaded DEGs to the online website DAVID to identify GO Terms and KEGG pathways and classified them into 3 functional categories: biological process (BP), cellular component (CC), and molecular function (MF; Figure 1A). As shown in Figure 1B and Table 2, GO analysis showed that the DEGs were most significantly enriched in muscle contraction and regulation of muscle contraction. Moreover, the upregulated DEGs were significantly enriched in biological process, including muscle system process, muscle contraction, and regulation of muscle contraction (Figure 1B and Table 2); the downregulated DEGs were enriched in organic acid transport, lipid metabolic process, and cellular lipid metabolic process (Figure 1B and Table 2).

**Table 2.** The Gene Ontology Analysis of DEGs Associated With Early-Onset Colorectal Cancer.

Category	Term	Count	*P* Value
Upregulated DEGs			
GOTERM_BP_FAT	GO:0003012∼muscle system process	23	2.84E-15
GOTERM_BP_FAT	GO:0006936∼muscle contraction	21	8.11E-15
GOTERM_BP_FAT	GO:0006937∼regulation of muscle contraction	12	1.87E-09
GOTERM_CC_FAT	GO:0043292∼contractile fiber	14	1.97E-09
GOTERM_BP_FAT	GO:0090257∼regulation of muscle system process	13	2.71E-09
GOTERM_CC_FAT	GO:0031012∼extracellular matrix	19	6.75E-09
GOTERM_CC_FAT	GO:0044449∼contractile fiber part	13	7.83E-09
GOTERM_BP_FAT	GO:0061061∼muscle structure development	19	1.41E-08
GOTERM_BP_FAT	GO:0001933∼negative regulation of protein phosphorylation	16	1.41E-08
GOTERM_BP_FAT	GO:0072358∼cardiovascular system development	23	3.91E-08
Downregulated DEGs			
GOTERM_BP_FAT	GO:0015849∼organic acid transport	4	4.75E-04
GOTERM_BP_FAT	GO:0006629∼lipid metabolic process	7	4.90E-03
GOTERM_BP_FAT	GO:0044255∼cellular lipid metabolic process	6	8.37E-03
GOTERM_BP_FAT	GO:0031667∼response to nutrient levels	4	1.36E-02
GOTERM_BP_FAT	GO:2000188∼regulation of cholesterol homeostasis	2	1.63E-02
GOTERM_BP_FAT	GO:0009991∼response to extracellular stimulus	4	1.66E-02
GOTERM_BP_FAT	GO:0010565∼regulation of cellular ketone metabolic process	3	1.67E-02
GOTERM_BP_FAT	GO:0031669∼cellular response to nutrient levels	3	1.69E-02
GOTERM_BP_FAT	GO:0007584∼response to nutrient	3	1.94E-02
GOTERM_BP_FAT	GO:0016137∼glycoside metabolic process	2	2.00E-02

Abbreviation: DEG, differentially expressed gene.

### Kyoto Encyclopedia of Genes and Genomes Pathway Analysis

**Figure 2.**
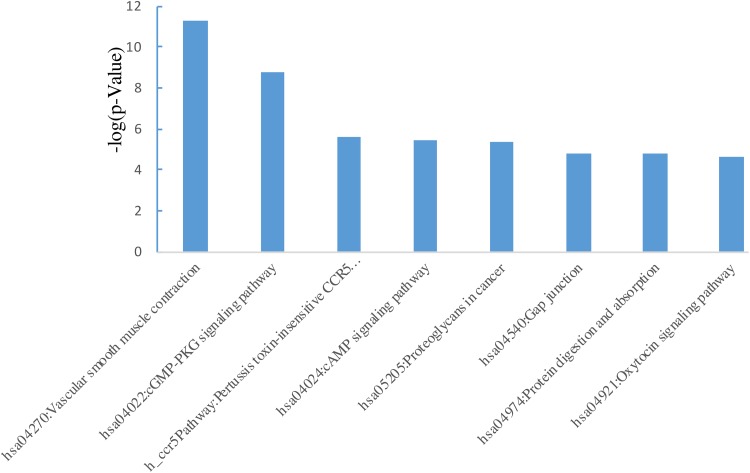
Significantly enriched signal pathway of differentially expressed genes (DEGs) in early-onset colorectal cancer (CRC).

We used online website DAVID to perform DEG functional and signaling pathway enrichment analysis. Figure 2 shows the most significantly enriched pathways of DEGs, and Table 3 lists the significantly enriched pathways of the upregulated DEGs, while there are no available significantly enriched pathways of the downregulated DEGs (Table 3). The significant signal pathway of the (upregulated) DEGs mainly enriched in vascular smooth muscle contraction and cGMP-PKG signaling pathway.

**Table 3.** Signaling Pathway Enrichment Analysis of DEGs Associated With Early-Onset Colorectal Cancer.

Pathway	Term	Count	*P* Value	Genes
Upregulated DEGs				
KEGG_Pathway: hsa04270	Vascular smooth muscle contraction	7	8.33E-05	*ACTA2, MYL9, ACTG2, GUCY1B3, KCNMB1, CALD1, GUCY1A3*
KEGG_Pathway: hsa04022	cGMP-PKG signaling pathway	7	5.12E-04	*MYL9, RGS2, ATP1A2, GUCY1B3, PLN, KCNMB1, GUCY1A3*
KEGG_Pathway: hsa05205:	Proteoglycans in cancer	6	0.007610	*FLNA, TIMP3, ANK2, DCN, FN1, CAV1*
BIOCARTA: h_ccr5Pathway	Pertussis toxin-insensitive CCR5 Signaling in Macrophage	3	0.012779	*FOS, CXCR4, CXCR12,*
KEGG_Pathway: hsa04921	Oxytocin signaling pathway	5	0.016252	*MYL9, GUCY1B3, FOS, GUCY1A3, RGS2*
KEGG_Pathway: hsa04540	Gap junction	4	0.017068	*GUCY1B3, TUBA1A, TUBB6, GUCY1A3*
Downregulated DEGs				
No significant signal pathway(*P* value <.05) available				

Abbreviation: DEG, differentially expressed gene.

### Module Analysis, Key Candidate Genes, and Pathway Identification From PPI Network

**Figure 3.**
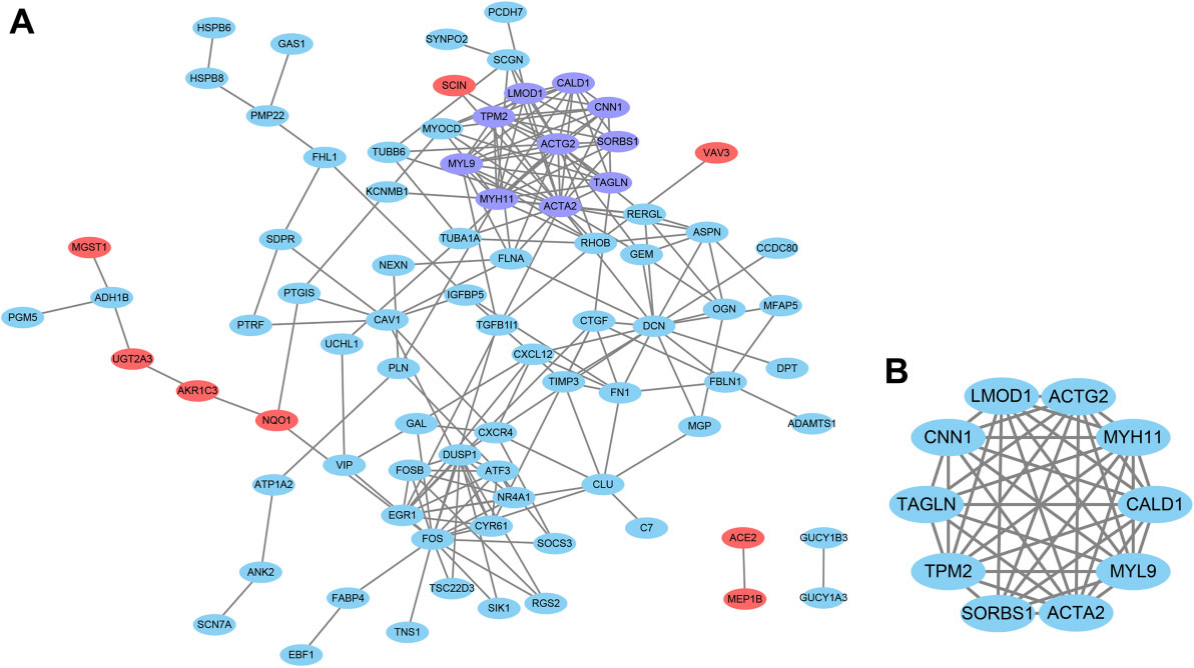
Protein–protein interaction (PPI) network of differentially expressed genes (DEGs; Node color: Skyblue indicates up-regulated gene, Red indicates down-regulated gene). (A) Based on the STRING online database, 82 DEGs were filtered into DEGs PPI network. (B) The most significant module from the PPI network.

Based on the STRING online database (http://string-db.org) and Cytoscape software, a total of 131 DEGs (108 upregulated and 23 downregulated genes) were filtered into the DEG PPI network complex, containing 82 nodes and 199 edges (Figure 3A), and 49 genes did not fall into the PPI network. According to the filtering of node degree ≥10 criteria, the top 10 hub genes were *ACTA2*, *ACTG2*, *FOS*, *DCN*, *MYH11*, *MYL9*, *EGR1*, *TPM2*, *LMOD1,* and *CALD1*. Based on the MCODE, the significant module (10 nodes 42 edges, Figure 3B) from the PPI network was selected, and the functional annotation of the common genes were analyzed (Table 4). Enrichment analysis showed that the genes were mainly associated with vascular smooth muscle contraction signaling pathway.

**Table 4.** Pathway Enrichment Analysis of Common Genes Function.

Term	Description	Count	FDR	Genes
GO.0006936	Muscle contraction	6	3.17E-07	*ACTG2, CALD1, LMOD1, MYH11, MYL9, TPM2*
GO.0043292	Contractile fiber	5	9.26E-06	*ACTA2, CALD1, LMOD1, MYH11, TPM2*
4270	Vascular smooth muscle contraction	4	1.87E-05	*ACTG2, CALD1, MYH11, MYL9*
GO.0030016	Myofibril	4	0.000315	*CALD1,LMOD1,MYL9,TPM2*
GO.0044449	Contractile fiber part	4	0.000315	*ACTA2,LMOD1,MYH11,TPM2*
GO.0016459	Myosin complex	3	0.000661	*ACTG2,MYH11,MYL9*
GO.0008307	Structural constituent of muscle	3	0.00147	*MYH11,MYL9,TPM2*
GO.0015629	Actin cytoskeleton	4	0.00264	*ACTG2,LMOD1,MYH11,TPM2*
GO.0005829	Cytosol	7	0.00274	*ACTA2,ACTG2,CALD1,LMOD1,MYH11,MYL9,TPM2*
GO.0005859	Muscle myosin complex	2	0.00477	*MYH11,MYL9*

## Discussion

The CRC is a disease of accumulated genetic, epigenetic, and environmental aberrations.^28^ Understanding the molecular mechanism of CRC is of very importance for diagnosis and treatment. It has been known that Wnt signaling pathway was associated with the major causes of CRC.

In this study, we expected to find out the key candidate genes and signal pathway in early-onset CRC. By comparing the 12 patients’ mucosa with 10 healthy control mucosa, 108 upregulated and 23 downregulated DEGs were screened. By using GO and PPI network analysis, 7 hub genes, namely, *ACTA2, ACTG2, MYH11, CALD1, MYL9, TPM2* and *LMOD1*, coupled with vascular smooth muscle contraction signaling pathway have been identified.

*ACTA2* (smooth muscle cell alpha actin) was identified as one of the hub genes showing the highest degree of connectivity. Lee’s group identified a correlation between early brain metastasis of lung adenocarcinoma and amplification of the *ACTA2* gene, and *ACTA2* could be a promising diagnostic and therapeutic target for lung cancer.^29^ The second hub gene *ACTG2* (actin gamma smooth muscle 2), encoding an ACTG2 protein, was related to metastasis of hepatocellular carcinoma.^30-33^ The third hub gene *MYH11* (myosin-11) is a smooth muscle myosin belonging to the myosin heavy-chain family.^34^ The *MYH11* gene may be related to cell migration and adhesion, intracellular transport, and signal transduction, and *MYH11* functions as a contractile protein, converting chemical energy into mechanical energy through adenosine triphosphate (ATP) hydrolysis. Wang et al^35^ reported that *MYH11* can contribute to predicting prognosis in stage II and III CRCs. Jo YS et al^36^ also reported an oncogenic fusion *CBFB/MYH11* and frameshift mutations in CRCs. Moreover, CALD1 (Caldesmon) encodes caldesmon protein, which is a calmodulin-binding and cytoskeleton-associated protein, and caldesmon is a biomarker for differentiation of smooth muscle.^37-39^ Yokota M group revealed that CALD1 showed a poor prognosis in colon cancer^40^ Myosin regulatory light polypeptide 9 (MYL9) encoded by MYL9 is a myosin light chain that may regulate muscle contraction by conducting the ATPase activity.^41^ The research unveiled that MYL9 expression level might be associated with the occurrence of non-small-cell lung cancer (NSCLC), which may be correlated to NSCLC metastasis.^42^ Another hub gene, TPM2 (β-Tropomyosin), encoded tropomyosin beta chain, which is roughly 32 KD in molecular weight.^43^ Bellavance^44^ suggested that TPM2 has an important role in regulating actin cable information and controlling actin nucleation in vivo. The last hub gene *LMOD1* (Leiomodin 1) is expressed in most tissue, with the high expression levels in thyroid, skeletal muscle, eye muscle, and ovary.^45^ Aberrant expression of *LMOD1* may be associated with the disease. Comley^46^ revealed that *LMOD1* was a novel component of the smooth muscle actin cytoskeleton.

Module analysis of the PPI networks suggested that the early-onset CRC is associated with vascular smooth muscle contraction signaling pathway, and the vascular smooth muscle cell (VSMC) principal function is contraction.^47^ The principal mechanisms that regulate the contractile state of VSMCs are changes in cytosolic Ca^2+^ concentration. Moreover, Rho/Rho kinase, PKC, and arachidonic acid have been proposed to play a pivotal role in this event.^48^

In conclusion, in this study, we investigated the potential candidate gene and signal pathway of DEGs in early-onset CRC. Genes were selected by DEG, GO, KEGG, and PPI analysis. This study has improved our understanding of the pathogenesis and underlying molecular mechanism in early-onset CRC; these selected candidate genes and pathways could give us a clue to new therapeutic targets for treatment of CRC. However, further molecular biological experiments are required to confirm the function of these identified genes in CRC.
