# Corrigendum

**DOI:** 10.1177/1073274819840084

**Published:** 2019-04-29

Zhao B, Baloch Z, Ma Y, et al. Identification of Potential Key Genes and Pathways in Early-Onset Colorectal Cancer Through Bioinformatics Analysis. Cancer Control. 2019; 26:1-8. DOI: 10.1177/1073274819831260

In this article, the authors have inadvertently forgotten to mention the below changes. These are now corrected in the article.

- 1st affiliation (Medical College of Xiamen University, Xiamen, Fujian, China) is incorrect. The correct affiliation is School of Medicine, Xiamen University, Xiamen, China.
- Author Bin Zhao is also affiliated with Department of Oncology and Vascular Interventional Radiology, Zhongshan Hospital, Xiamen University, Xiamen, China.
- Corresponding author Yilin Zhao, School of Medicine, Xiamen University, Xiamen, China. Email: zyllbz@gmail.com

